# Epigenetic Regulation of DLK1-DIO3 Region in Thyroid Carcinoma

**DOI:** 10.3390/cells13121001

**Published:** 2024-06-08

**Authors:** Letícia F. Alves, Isabelle N. da Silva, Diego C. de Mello, Cesar S. Fuziwara, Sonia Guil, Manel Esteller, Murilo V. Geraldo

**Affiliations:** 1 Josep Carreras Leukaemia Research Institute, 08916 Barcelona, Spain; lferreira@carrerasresearch.org (L.F.A.);; 2 Department of Structural and Functional Biology, University of Campinas (UNICAMP), Sao Paulo 13083-863, Brazil; 3 Department of Cell and Developmental Biology, Institute of Biomedical Sciences, University of Sao Paulo, Sao Paulo 05508-000, Brazil

**Keywords:** thyroid cancer, epigenetics, methylation, ncRNAs

## Abstract

Non-coding RNAs (ncRNAs) have emerged as pivotal regulators in cellular biology, dispelling their former perception as ‘junk transcripts’. Notably, the DLK1-DIO3 region harbors numerous ncRNAs, including long non-coding RNAs (lncRNAs) and over 50 microRNA genes. While papillary thyroid cancer showcases a pervasive decrease in DLK1-DIO3-derived ncRNA expression, the precise mechanisms driving this alteration remain elusive. We hypothesized that epigenetic alterations underlie shifts in ncRNA expression during thyroid cancer initiation and progression. This study aimed to elucidate the epigenetic mechanisms governing DLK1-DIO3 region expression in this malignancy. We have combined the analysis of DNA methylation by bisulfite sequencing together with that of histone modifications through ChIP-qPCR to gain insights into the epigenetic contribution to thyroid cancer in cell lines representing malignancies with different genetic backgrounds. Our findings characterize the region’s epigenetic signature in thyroid cancer, uncovering distinctive DNA methylation patterns, particularly within CpG islands on the lncRNA MEG3-DMR, which potentially account for its downregulation in tumors. Pharmacological intervention targeting DNA methylation combined with histone deacetylation restored ncRNA expression. These results contribute to the understanding of the epigenetic mechanisms controlling the DLK1-DIO3 region in thyroid cancer, highlighting the combined role of DNA methylation and histone marks in regulating the locus’ expression.

## 1. Introduction

Thyroid carcinoma is the most prevalent endocrine cancer worldwide. From the 2000s until recently, it had the fastest-growing incidence in the U.S., primarily attributed to advancements in diagnostic techniques, which resulted in cases of overdiagnosis. However, due to the adoption of more conservative diagnostic criteria, the incidence rate has been declining by approximately 2% per year since 2014 [1]. Well-differentiated carcinomas are the prevalent form of malignant tumors of the thyroid gland, and they include the papillary subtype (PTC), which represents about 85% of all cases of thyroid cancer (TC) in the U.S., and follicular carcinoma (FTC), accounting for approximately 5% of the cases. Undifferentiated carcinomas are represented by anaplastic thyroid carcinoma (ATC), the least prevalent (<2% of the cases) but the one that presents the worst prognosis [2].

The aberrant levels of ncRNAs found in thyroid tumor samples and the link of these molecules with classic oncogenes and tumor suppressors highlighted this subtype of RNA as a promising therapeutic target [3,4,5]. The delta-like non-canonical Notch ligand 1/iodothyronine deiodinase 3 (DLK1-DIO3) region is located in chromosome 14 and hosts the largest miRNA cluster of the human genome. This region is highly conserved and presents more than 50 miRNA genes, which give rise to over 100 mature miRNAs [6]. The large-scale analysis of miRNA expression in the PTC murine model revealed the global downregulation of several miRNAs situated in the DLK1-DIO3 genomic region [7]. Importantly, the restoration of a single miRNA from the DLK1-DIO3 region induced the decrease in both cell migration and invasion of the tumor cells in vitro, in addition to the restoration of the expression of tumor suppressors, suggesting a tumor suppressor role for miRNAs from this region [8]. The bioinformatics construction of a post-transcriptional regulation network potentially regulated by the DLK1-DIO3-miRNAs revealed that these molecules might take part in important processes for TC progression, such as cell adhesion and migration, angiogenesis, and extracellular matrix remodeling [9].

The coordinated downregulation of DLK1-DIO3-derived miRNAs in human PTC samples [7] reinforces the polycistronic nature of the region, and its control is closely linked to epigenetic mechanisms [10,11,12,13,14]. Acting in the maintenance of the region’s imprinting are two main differentially methylated regions (DMRs). Even though these regions have been vastly explored in healthy and disease contexts, the alterations in the epigenetic marks controlling this region in TC development and progression are still elusive. We hypothesize that the modification of the DNA methylation pattern in these DMRs could be involved in the decreased expression of DLK1-DIO3-derived ncRNAs in TC.

## 2. Materials and Methods

### 2.1. Cell Culture

A panel of four thyroid-derived cell lines was used in this study. NThy-ORI is a normal follicular immortalized cell line; TPC-1 and BCPAP cell lines are derived from human papillary carcinoma and harbor the RET/PTC1 chromosomal rearrangement and the BRAFT1799A mutation, respectively; KTC-2 is derived of human anaplastic carcinoma and also harbors the BRAFT1799A mutation. NThy-ORI was donated by Dr. Edna Kimura from the University of Sao Paulo, Brazil. The TPC-1 cell line was provided by Dr. James A. Fagin (Human Oncology and Pathogenesis Program, Memorial Sloan-Kettering Cancer Center, New York, NY, USA). The BCPAP cells were donated by Dr. Massimo Santoro (Medical School, University ‘Federico II’ of Naples, Naples, Italy). NThy-ORI cells were maintained in RPMI medium supplemented with 10% fetal bovine serum (SFB, Thermo Fisher, Waltham, MA, USA) and 2 nM of L-glutamine. TPC-1 and BCPAP were maintained in DMEM medium supplemented with 5% and 10% fetal bovine serum, respectively. KTC-2 cell line was maintained in RPMI medium supplemented with 10% fetal bovine serum. All cell lines were maintained in a 37 °C incubator with 5% of CO_2_ with antibiotics (penicillin 100 U/mL and streptomycin 100 μg/mL, Thermo Fisher, Waltham, MA, USA) and antifungal (amphotericin B 1 μg/mL, Thermo Fisher, Waltham, MA, USA).

### 2.2. Methylation Array

Illumina’s Infinium MethylationEPICBeadChip (Illumina, San Diego, CA, USA) was performed in collaboration with the Genomics Unit at Josep Carreras Research Institute using the Illumina HiScanTM SQ fluorescent scanner and the Freedom EVO platform (Tecan Trading AG, Männedorf, Switzerland). EPIC array data were analyzed using limma, minfi, and DMRcate packages on R [15,16,17,18]. Briefly, data were normalized and filtered, and b-values and m-values were generated for further data visualization and statistical analysis. CpGs were considered differentially methylated positions (DMPs) when q-values < 0.05 and absolute differences between compared groups were ≥0.8.

### 2.3. MAPK Pathway Inhibition

MAPK pathway inhibition was performed using the pharmacological agent PD98059 (Thermo Fisher, Waltham, MA, USA), which is an MEK1 inhibitor. For such, 5 × 10^5^ cells were seeded in 60 mm plates and, after 24 h, treated with 20 μM of the inhibitor (reconstituted in DMSO). The control group was treated with DMSO. After 24, 48, or 72 h of treatment, the media was removed, and cells were washed with PBS and collected in 1 mL of TRIzol for RNA extraction.

### 2.4. TCGA Methylation Data Analysis

Methylation data from the TCGA (The Cancer Genome Atlas) THCA project were downloaded using the SMART platform (accessed on 7 July 2023) [19] and further analyzed or processed for data visualization using R programming language. The data comprised 56 normal (healthy) thyroid samples and 511 thyroid tumor samples. Chromatin state segmentation was assessed using ENCODE/Broad data by HMM [20]. Prediction of transcription factor binding was performed using Alibaba2 http://gene-regulation.com/pub/programs/alibaba2/ (accessed on 20 September 2023) with default parameters [21].

### 2.5. Bisulfite-qPCR

Genomic DNA was isolated by lysing cell pellets with ProK/Lysis buffer (2 mg/mL proteinase K, 50 mM Tris–HCl, pH8.0, 0.1 M NaCl, 20 mM EDTA, 1% SDS) at 56 °C overnight. DNA samples were quantified and assessed for purity by NanoDrop-1000 Spectrophotometer (Thermo Scientific, Waltham, MA, USA) 260/280 and 170 260/230 ratio measurements. DNA integrity was checked by electrophoresis in a 2% agarose gel. One microgram of purified genomic DNA was submitted to bisulfite conversion using the EZ DNA-methylation gold kit (Zymo Research, Irvine, CA, USA), following the manufacturer’s instructions. Next, samples went through PCR for the amplification of target regions (Table S1). PCR reactions were applied to a 2% agarose gel to isolate fragments, and the desired bands were purified using NucleoSpin Gel and PCR Clean-up (Macherey-Nagel, Düren, Germany), ligated to the pGEM-T Easy plasmid (Promega, Madison, WI, USA) and transformed to DH5α competent bacteria. Fourteen to sixteen clones of each construction were picked, grown, and then submitted to a mini-prep using NucleoSpin 96 Plasmid (Macherey-Nagel, Düren, Germany). Constructions were then sequenced on the ABI Prism 3130XL Applied Biosystems DNA sequencer to identify methylated CpGs.

### 2.6. Overall Survival Analysis

Methylation and clinical data from the THCA project were downloaded from GDC using the TCGAbiolinks R package (version 2.30.4) [22]. M- and B-values were calculated using limma (version 3.58.0) [17], and individual values were compared to samples’ b-value means to identify samples with higher or lower methylation profiles. Kaplan–Meier curves were calculated and plotted using survival (version 3.6-4) and survminer (version 0.4.9) packages [23,24]. Data on other types of cancer were retrieved from MethSurv (accessed on 22 March 2024) [25].

### 2.7. Treatment with 5-Aza-dC and TSA 

Cell lines were seeded (3 × 10^5^) in 100 mm plates, and after 24 h, 5μM of 5-Aza-dC (A3656-5MG—Sigma-Aldrich, St. Louis, MO, USA) was added to the appropriate culture medium followed by incubation at 37 °C for 72 h. The culture medium was changed to a fresh medium containing the same concentration of 5-Aza-dC every 24 h. For trichostatin A (TSA, T8552-5MG—Sigma-Aldrich, St. Louis, MO, USA) treatment, cells were cultivated for 48 h, and TSA in a concentration of 300 nM was added 24 h before RNA collection. For combined treatment, the cells were treated with 5-Aza-dC (5 μM) for 48 h and then TSA (300 nM) for another 24 h before RNA collection. After the treatment, RNA extraction was performed using Maxwell RSC miRNA Tissue Kit (Promega, Madison, WI, USA) and following the manufacturer’s instructions.

### 2.8. Quantitative Reverse Transcription PCR (RT-qPCR)

cDNA synthesis was performed according to Invitrogen’s M-MLV instructions with 1 μg of total RNA per reaction. For RT-qPCR reactions, we used 10 μL of ThermoFisher’s SYBR Master Mix, 5 μL of the pair of primers (MEG3_F ‘5-GCATTAAGCCCTGACCTTTG-3′, MEG3_R 5′-TCCAGTTTGCTAGCAGGTGA-3′), and 5 μL of diluted cDNA. The reactions were performed in the Applied StepOne Plus thermocycler, and the ThermoCloud platform (Thermo Scientific, Waltham, MA, USA) was used to analyze data.

### 2.9. Chromatin Immunoprecipitation (ChIP) for Histone Modifications

ChIP analysis of histone modifications was performed on formaldehyde crosslinked chromatin isolated from 15 × 10^6^ cells for all cell lines analyzed. Briefly, chromatin extraction and shearing were performed following the high cell protocol from the TruChIP Chromatin Shearing Kit (PN 520154—Covaris, Woburn, MA, USA). Sixty micrograms of sheared chromatin were diluted with the IP buffer and pre-cleared with non-immune rabbit IgG and protein A magnetic beads (Dynabeads, Thermo Scientific, Waltham, MA, USA) for 2 h at 4 °C on a rotating wheel. Five micrograms of anti-histone modification antibodies (pan histone H3, H3K27ac, H3K9me3) or normal rabbit IgG were added to pre-cleared chromatin and incubated overnight at 4 °C on a rotating wheel. The pan histone H3 antibody and IgG were used as experimental controls. Chromatin was precipitated overnight at 4 °C with rotation. The beads were then washed in three different buffers and TE. The bound chromatin was eluted into an elution buffer, followed by crosslink reversal and protein digestion. DNA was extracted by phenol/chloroform and ethanol precipitated in the presence of 10 μg of glycogen. The obtained DNA was then used to perform real-time qPCR reactions (primers shown in Table S2).

## 3. Results

### 3.1. DNA Methylation of the DLK1-DIO3 Region Is Disrupted in Thyroid Cancer-Derived Cell Lines

**Figure 1:**
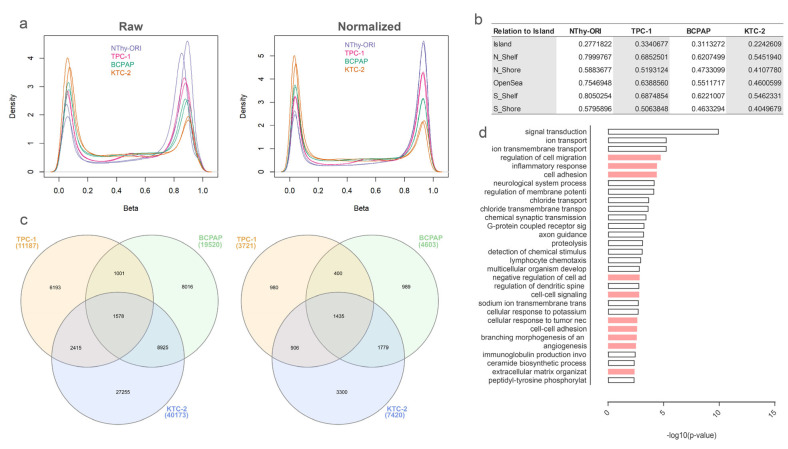
DNA methylation in thyroid cancer cell lines. (**a**) B-values reveal a decreasing pattern of methylation across TC samples. The plots show the distribution of B-values before (**left**) and after (**right**) normalization. (**b**) Table of mean b-values in regions related to CpG island for each cell line. (**c**) Differential DMP genes across tumor cell lines compared to a non-tumoral cell line. The Venn diagram on the left shows the distribution of differential DMPs across each cell line and its intersections. The Venn diagram on the right shows the distribution of the genes with differential DMPs across each cell line and its intersections. (**d**) Functional enrichment of differential DMP genes common between BCPAP and KTC-2. The plot shows the top 30 enriched biological processes for the pool of differential DMP genes shared between BCPAP and KTC-2. Categories related to cancer progression are shown in pink bars.

Given its extension, the methylation status of the DLK1-DIO3 region in thyroid-derived cell lines was accessed by Illumina’s EPIC methylation array (Figure 1a). The global methylation levels followed the cell line differentiation status, with highly differentiated cells showing a more methylated profile (higher density of higher b-values) and decreasing across the TC cell lines (Figure 1a). The higher global methylation levels were found in the normal immortalized cell line (NThy-ORI), followed by the PTC-derived cell line harboring the RET/PTC rearrangement (TPC-1), the PTC-derived cell line, which harbors the BRAFT1799A mutation (BCPAP), and the ATC-derived cell line, which also harbors the BRAFT1799A mutation (KTC-2). Mean b-values for each cell line according to regions in relation to CpG islands are displayed in Figure 1b. DMPs were identified and compared for the contrasts between the normal and tumoral cell lines (Figure 1c left). The genes where the DMPs were found were also considered (Figure 1c right), and the functional enrichment of the pool of genes from the intersection of BCPAP and KTC-2 revealed categories related to cancer progression and aggressiveness (Figure 1d).

**Figure 2:**
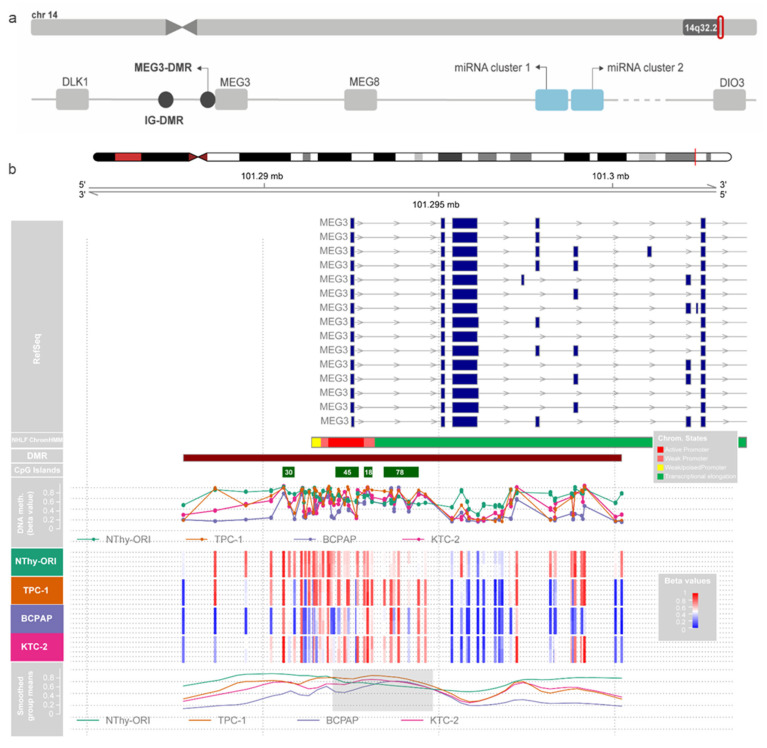
Methylation in the MEG3-DMR region. (**a**) Schematic representation of the imprinted DLK1-DIO3 region. (**b**) The figure shows methylation b-values for each cell line in duplicates for the DLK1-DIO3 region. Tracks are as follows, from top to bottom: chromosome 14 representation, RefSeq annotated genes, algorithm-predicted DMR, CpG Islands from UCSC, methylation b-values for each cell line, and the lines of smoothed group means.

The analysis of the CpGs located on the DLK1-DIO3 region revealed a highly methylated profile of this region in the non-tumoral cells. In contrast, the tumoral cell lines showed different degrees of hypomethylation (Figure S1). The algorithm-based prediction of DMRs was performed using the dmrcate function (DMRcate version 1.8.6), which computes a kernel estimate against a null comparison to identify significantly differentially methylated regions. This step revealed a DMR containing four CpG islands (CGIs) overlapping the lncRNA *MEG3* promoter and 5′ region (DMRs are represented by filled circles in Figure 2a). From now on, these CGIs will be referred to by the number of contained CpGs according to the genome annotation hg38 (e.g., CGI 30, CGI 45, CGI 18, and CGI 78). Interestingly, when we observed the smoothed means of b-values of the region containing these CGIs, we noticed that, in a particular extension of the MEG3-DMR, the methylation levels are higher in the tumor cell lines than in the normal cell line, contrasting the data obtained for the region as a whole (Figure 2b). We did not find algorithm-predicted hypermethylated DMRs on tumoral cell lines in the IG-DMR, the intergenic region upstream of *MEG3*. 

**Figure 3:**
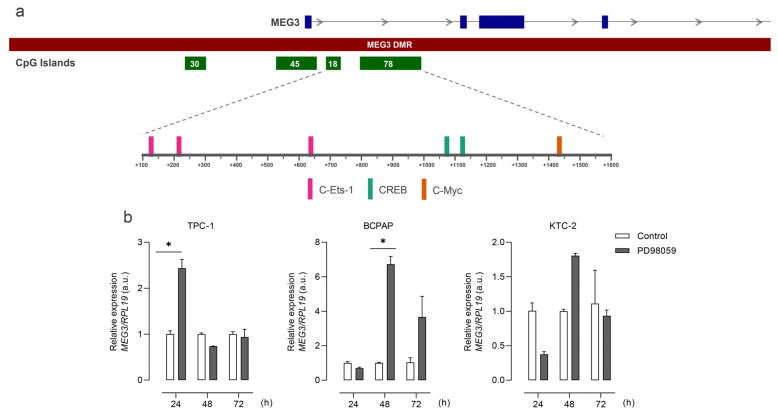
MAPK TF binding sites are present in MEG3-DMR, and MAPK signaling modulates MEG3 expression. (**a**) Scheme of TF binding sites for MAPK pathway downstream effectors on MEG3-DMR. (**b**) Bar plots show MEG3 expression for the four cell lines in response to the treatment with the MEK inhibitor PD98059. *: *p* < 0.05.

We then accessed the data from the Chromatin State Segmentation by HMM from ENCODE/Broad and searched for the presence of regulatory elements throughout this region in different models. The region where these CGIs are placed corresponds to an active promoter in the HUVEC and NHFL cell lines (Figure 2b). Additionally, an algorithm-based prediction identified 189 segments in the sequence of the hypermethylated CpGs as potential binding sites for known transcription factors. Among these sites, we highlight three sites for C-Ets-1, two for Creb, and one for c-Myc, important downstream effectors of the MAPK pathway (Figure 3a). Interestingly, the treatment of the panel of thyroid cell lines with a MEK inhibitor (PD98059) had fluctuating effects on rescuing the expression of MEG3 at different time points (Figure 3b), indicating that the MAPK pathway status could be related to MEG3 expression control (or even the expression of the region as a whole). This variability suggests that the pathway impacts expression, even if it does not consistently proceed in one direction.

### 3.2. Hypermethylation of MEG3-DMR CGIs in Human Papillary Thyroid Carcinoma Samples from TCGA

**Figure 4:**
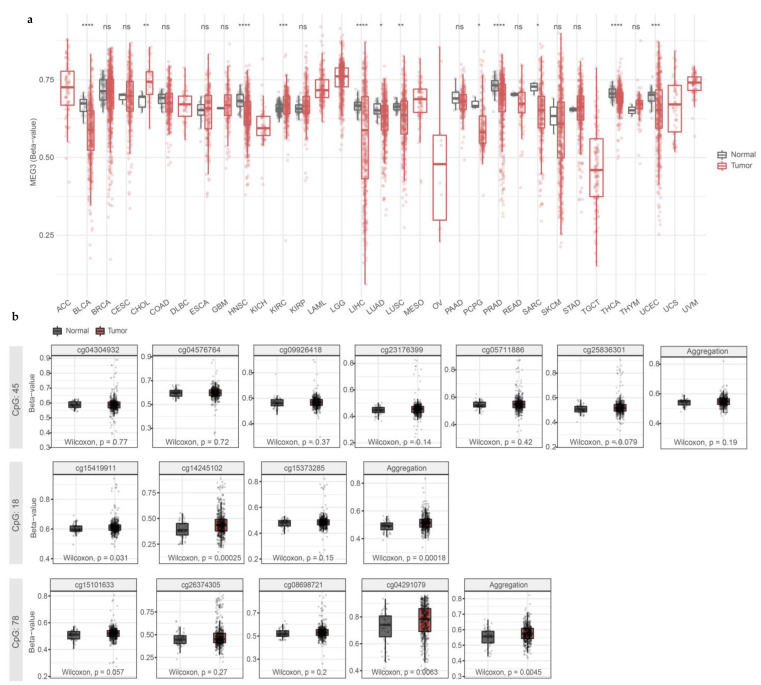
DNA hypermethylation in the *MEG3* promoter region is restricted to CGI 18 and 78 in human thyroid cancer samples. (**a**) *MEG3* methylation is overall decreased in the thyroid tumor samples. (**b**) Methylation in CGIs 45, 18, and 78 in normal and tumor thyroid samples. ns: *p* < 0.05; *: *p* < 0.05; **: *p* < 0.01; ***: *p* < 0.001; ****: *p* < 0.0001. (ACC) Adrenocortical carcinoma; (BLCA) Bladder Urothelial Carcinoma; (BRCA) Breast invasive carcinoma; (CESC) Cervical squamous cell carcinoma and endocervical adenocarcinoma; (CHOL) Cholangiocarcinoma; (COAD) Colon adenocarcinoma; (DLBC) Lymphoid Neoplasm Diffuse Large B-cell Lymphoma; (ESCA) Esophageal carcinoma; (GBM) Glioblastoma multiforme; (HNSC) Head and Neck squamous cell carcinoma; (KICH) Kidney Chromophobe; (KIRC) Kidney renal clear cell carcinoma; (KIRP) Kidney renal papillary cell carcinoma; (LAML) Acute Myeloid Leukemia; (LGG) Brain Lower-Grade Glioma; (LIHC) Liver hepatocellular carcinoma; (LUAD) Lung adenocarcinoma; (LUSC) Lung squamous cell carcinoma; (MESO) Mesothelioma; (OV) Ovarian serous cystadenocarcinoma; (PAAD) Pancreatic adenocarcinoma; (PCPG) Pheochromocytoma and Paraganglioma; (PRAD) Prostate adenocarcinoma; (READ) Rectum adenocarcinoma; (SARC) Sarcoma; (SKCM) Skin Cutaneous Melanoma; (STAD) Stomach adenocarcinoma; (TGCT) Testicular Germ Cell Tumors; (THYM) Thymoma; (THCA) Thyroid carcinoma; (UCS) Uterine Carcinosarcoma; (UCEC) Uterine Corpus Endometrial Carcinoma; (UVM) Uveal Melanoma.

Next, we looked at the methylation data from the THCA dataset from the TCGA consortium. In human tumor samples, we found that the *MEG3* gene was generally hypomethylated (Figure 4a), except for the particular CGIs inside MEG3-DMR, with significant hypermethylation in cg15419911 and cg14245102 (CGI 18) and cg04291079 (CGI 78) (Figure 4b).

**Figure 5:**
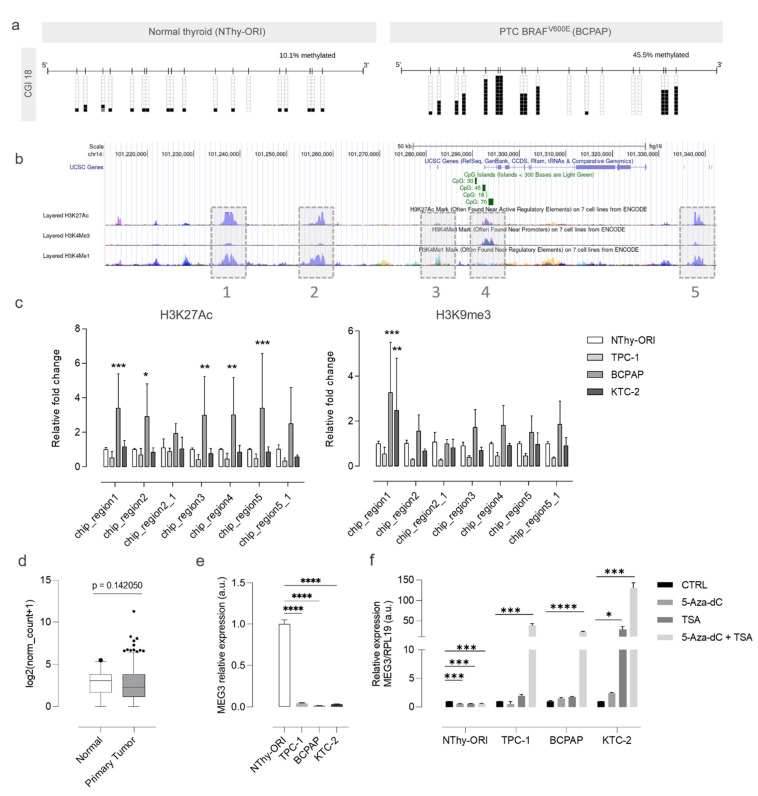
Epigenetic regulation of *MEG3* expression in thyroid cancer. (**a**) Plots show the methylation status of CGI 18 in NThy-ORI (non-tumoral) and BCPAP (tumoral) cell lines. Methylated CpGs are represented in black-filled squares, unmethylated CpGs are represented in blank squares, and hatched boxes represent cytosine residues with unknown methylation status. (**b**) The figure shows part of the DLK1-DIO3 region with the target regions for ChIP highlighted in gray boxes. Given the extension of the chosen regions, some were covered by two amplicons annotated using the underscore (e.g., 2 and 2_1, 5 and 5_1). (**c**) Bar plots show the relative fold-change of histone marks (H3K27Ac on the left and H3K9me3 on the right) in the different thyroid cell lines. Data are presented as the average of duplicates of two independent experiments, and bars represent the standard deviations (SDs). (**d**,**e**) The plots show MEG3 expression across thyroid cancer samples from TCGA and thyroid cancer cell lines. (**f**) The bar plot shows the expression of MEG3 after the individual or combined treatment with 5 uM 5-Aza-dC and 300 nM TSA. Data are presented as the average of triplicates of two independent experiments, and bars represent the standard deviations (SDs). *: *p* < 0.05; **: *p* < 0.01; ***: *p* < 0.001; ****: *p* < 0.0001.

To validate the above-cited findings, we performed the bisulfite-PCR analysis of the CGIs situated in MEG3-DMR in the normal thyroid-derived cell line NThy-ORI and the BRAF-mutated BCPAP, which resemble aggressive features observed in patients with poorly differentiated carcinomas. We targeted two regions on the IG-DMR as a comparison factor outside the MEG3-DMR. The analysis of the methylation status of these regions revealed differences between NThy-ORI and BCPAP cells. BCPAP cells showed a strong demethylated phenotype, especially in the IG-DMR and CGI 30 regions (Figure S2). On the other hand, CGI 18 showed hypermethylation in the tumor cell line (Figure 5a).

Crossing survival data with methylation levels of the probes on *MEG3* CGIs pointed to CpGs whose methylation status could be important for disease outcome. We could not find any significant association between individual CpG methylation and overall survival in thyroid cancer patients. However, we further looked at the 3 CpGs with lower *p*-values (Figure S3) in other types of cancer and found them to be significantly correlated to survival in cancers with lower survival rates. Cg05711886 was significatively (likelihood test *p*-value < 0.05) correlated to survival probability in five types of cancer, cg14123427 in six, and cg15373285 in nine (Table S3). Such information indicates that the methylation status of this region’s CpGs could have biomarker potential for other malignancies and should be further investigated. 

### 3.3. Silencing of MEG3 Is Mediated by DNA Methylation and Histone Acetylation

DNA and histone modifications often work in concert, and thus, we next explored the influence of histone modifications on the expression of MEG3. We performed ChIP assays for key histone modification marks to investigate the ones involved in the silencing of *MEG3*. The regions and histone marks chosen for the analysis were decided based on the information provided by previous studies [11,12,21] (Figure 5b) The analysis revealed a significant increase in the relative occurrence of H3K27Ac, a mark normally associated with positive regulation, in BCPAP cells (Figure 5c). For H3K9me3, a mark associated with silencing, a significant increase was found for BCPAP and KTC-2 only in chip region 1 (Figure 5c).

The expression of *MEG3* is slightly reduced in thyroid tumor samples and drastically reduced or abolished in thyroid tumor cell lines (Figure 5d,e). The expression of *MEG3* in the tumor cells could not be restored by 5-Aza-dC therapy alone (Figure 5f). The combined treatment (5-Aza + trichostatin A) successfully restored MEG3 expression in all tumor cells, indicating that the gene is controlled by both methylation and histone alterations (Figure 5f).

## 4. Discussion

The abnormal expression of coding and non-coding genes from the DLK1-DIO3 region has been extensively reported in different disease contexts. We have previously identified the global downregulation of DLK1-DIO3-derived miRNAs in papillary thyroid carcinoma [7]. This work sheds light on the epigenetic mechanisms controlling the transcription of DLK1-DIO3-derived ncRNA in thyroid cancer (TC), indicating an interplay between histone marks and DNA methylation in regulating the transcriptional state of the DLK1-DIO3 region.

The epigenetic regulation of the DLK1-DIO3 region has been frequently attributed to two differentially methylated regions (DMRs) within the locus: the intergenic DMR (IG-DMR) and the MEG3-DMR. IG-DMR, which is the imprinting control region (ICR) of the whole DLK1-DIO3 locus, has been the most explored in the literature [26]. In healthy somatic cells, this DMR is usually hypomethylated on the maternal allele and hypermethylated on the paternal allele [27], and this state has been shown to relate to the activation of an enhancer by controlling the binding of AFF3 [11].

The MEG3-DMR is a secondary DMR, starting approximately 1.5 kb upstream of the MEG3 gene and extending into the MEG3 intron 1 [28,29,30]. Zhu et al. have demonstrated that the maternal deletions of MEG3 exons 1–4 cause LOI (loss of imprinting), but the same is not observed for deletions of exons 2–4, indicating that the MEG3-DMR regulates imprinting [31]. Accordingly, the methylation status of MEG3-DMR has been associated with the expression of the DLK1-DIO3-derived miRNAs [32].

Global methylation analysis in thyroid-derived cell lines revealed hypomethylation correlating with differentiation status, consistent with previous studies associating global DNA hypomethylation with tumor grade and stage [33]. The analysis of methylation in the DLK1-DIO3 region revealed the hypermethylation in two CGIs in the MEG-DMR in the tumoral cell lines, suggesting a possible mechanism for the observed silencing of the region in the TC context. Global hypomethylation, together with the focal hypermethylation of regulatory elements associated with CGIs, is constantly seen in cancer [34].

It is important to consider that profiling cell lines may not directly reflect the landscape of the deriving tumors. It has been shown that cell lines derived from differentiated thyroid cancers display mRNA expression profiles closer to dedifferentiated in vivo thyroid tumors (i.e., ATC) than to differentiated ones [35,36]. To ensure that the findings were not restricted to the cell lines, we analyzed methylation data generated by the TCGA consortium for thyroid cancer (THCA project). The hypermethylation of the above-cited CGIs was confirmed in the dataset.

In concordance with the global downregulation of the DLK1-DIO3 region previously reported, we observed a pronounced suppression of MEG3 expression in the tumor cell lines. Loss of MEG3 expression has been reported to cause tumors and impact processes such as cell proliferation, apoptosis, and metastasis [37,38,39]. The findings from the global demethylation assay further support the notion that hypermethylation of MEG3-DMR CGIs could, at least in part, influence the region’s expression, as evidenced by the restored expression of these lncRNAs in tumor cell lines post-treatment. Notably, only the combination of 5-Aza + TSA effectively reactivates MEG3, suggesting that histone modifications also contribute to regulating MEG3 expression. Considering the ChIP results, region 1 emerges as significant, exhibiting heightened silencing marks in BCPAP and KTC-2 despite being distant from CGI18. Nonetheless, it is plausible that these two factors synergistically dictate transcriptional control.

Further efforts are needed to achieve a higher resolution characterization of histone marks in the DLK1-DIO3 region and pinpoint the key regulatory players in TC. Additionally, investigating these mechanisms in other thyroid cancer models, such as in vivo and human PTC samples, could help determine whether the findings are promising prospects for therapeutic approaches.

## 5. Conclusions

Overall, our results are an important contribution to comprehending the epigenetic mechanisms controlling the expression of the DLK1-DIO3-derived ncRNAs in TC. In this context, the methylation status of MEG3-DMR rather than IG-DMR determines the expression of the *MEG3* locus.

## Data Availability

The results shown here are partly based upon data generated by the TCGA Research Network: https://www.cancer.gov/tcga (accessed on 7 July 2023).

## Supplementary Materials

The following supporting information can be downloaded at https://www.mdpi.com/article/10.3390/cells13121001/s1, Table S1: Bisulfite-PCR primers, Figure S1: Methylation of DLK1-DIO3 region, Table S2: ChIP primers, Figure S2: TF binding sites for MAPK pathway downstream effectors on MEG3-DMR, Figure S3: Kaplan–Meier plots of the probes with lower *p*-values in THCA, Table S3: Survival data for top 3 CpGs.
