# Supplementary material for: Epigenetic Regulation of DLK1-DIO3 Region in Thyroid Carcinoma
Source: Cells. 2024 Jun 8;13(12):1001. doi: 10.3390/cells13121001 (PMC11201930; doi:10.3390/cells13121001)
Supplement: Supplementary file 1 [file cells-13-01001-s001.zip › cells-3007558-supplementary.pdf]

Epigenetic Regulation of DLK1-DIO3 Region in Thyroid Carcinoma

Supplementary Material

Table S1. Bisulfite-PCR primers

Region	Forward	Reverse
IG-DMR_1	TTTTATTATTGAATTGGGTTTGTTAGT	ACAATTCCTACTACAAAATTTCAACA
IG-DMR_2	GTTAAGAGTTTGTGGATTTGTGAGAAATG	CTAAAAATCACCAAAACCCATAAAATCAC
CGI 30	TTGGTATTGTGTTTAYGATAGTTT	AAAACRTTTATTAACCACAATATTAA
CGI 45_1	YGGTTAATTATTTTTAGAGAAATG	CCAAAATCAAACAAACTCTC
CGI 45_2	TTGTGTTTGAATTTATTTTGTTT	CCCCAAATTCTATAACAAATTACT
CGI 18	AGGAATTAGATGGAATTTTTTTT	CTAAAATCCACACTACACTAAACC
CGI 78_1	TAGGTTTTTGGAAGGTTTTTT	ACTAACCTTATCACAACCTCTCTC
CGI 78_2	GATTTTTGTTTTTTTTTGTAGTAGG	CAACTCAAAACCCAAAATAAC

Table S2. ChIP primers.

Name	Sequence	Amplicon size
ChIP_region1_F	CGGGCTCTCTCTTGTGGTTA	164
ChIP_region1_R	GCTGAGCTGCACACCTACTG	
ChIP_region2_F	ACAGGCATGGACAGAAGGTC	238
ChIP_region2_R	CGGGGCTATTTTAGGTGTCA	
ChIP_region2_1_F	CCTGATCCCCAAGTCTGAAA	218
ChIP_region2_1_R	TTTGCCATCTTCCTCATTCC	
ChIP_region3_F	GGGAGGCCCCTCAGTATAAA	226
ChIP_region3_R	AGGCCCTGAGAGTGAGTGAA	
ChIP_region4_F	TGGAACTCCTCTTGGATTCG	164
ChIP_region4_R	ACCCTTCTATTCGGGTGCTT	
ChIP_region5_F	TTTTGTCCCTCCCCAATGTA	202
ChIP_region5_R	AAAGCGGAGAAAGGAGGTTC	
ChIP_region5_1_F	CTGTCCTCTGCGTGTGTGTC	183
ChIP_region5_1_R	TCAGGGAAGGGCTCTGAGTA	

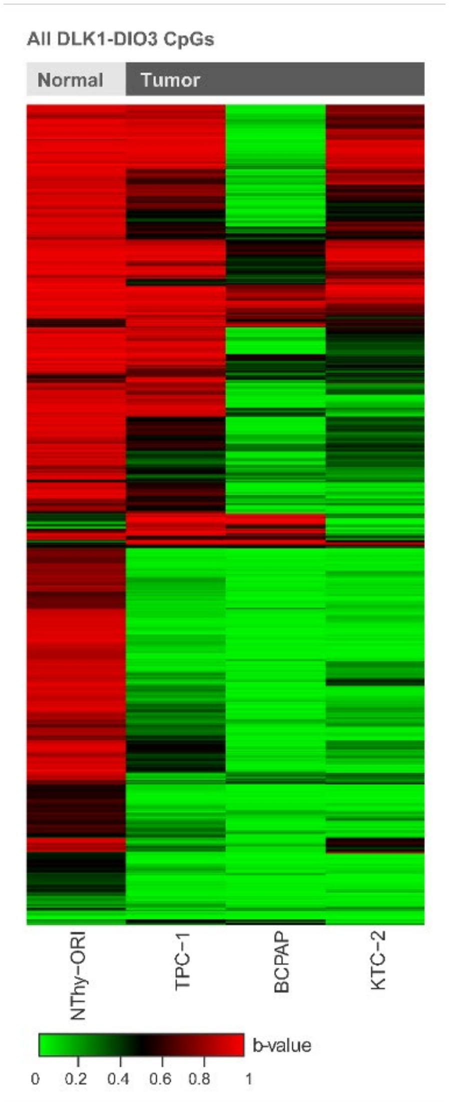

Figure S1. Methylation of DLK1-DIO3 region. Heatmap shows b-values for the DLK1-DIO3 CpGs in our panel of cell lines.

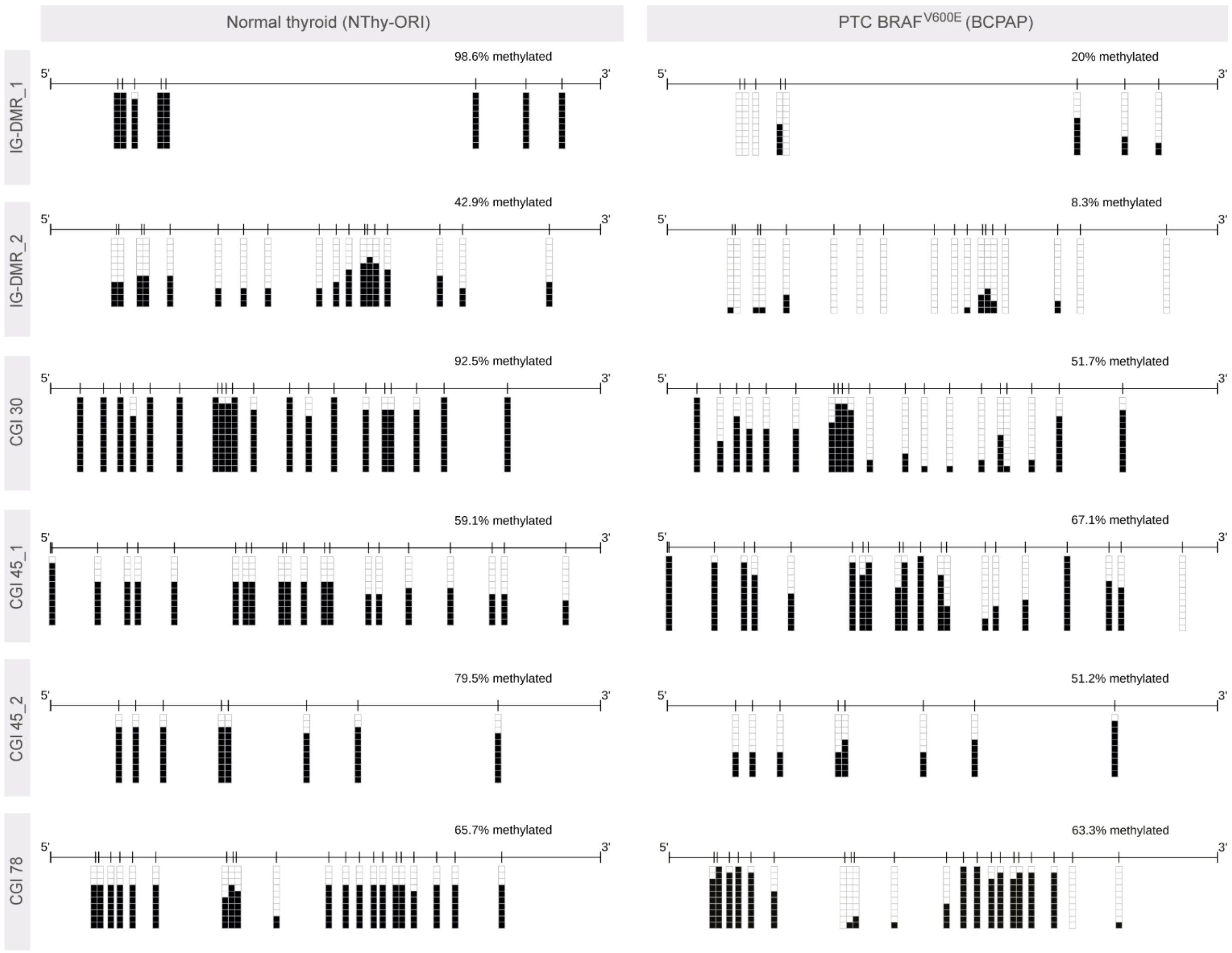

Figure S2. Methylation status of IG- and MEG3- DMRs in normal and tumor thyroid cells. Plots show the methylation status of investigated regions on IG- and MEG3- DMRs. Methylated CpGs are represented in black-filled squares and unmethylated CpGs are represented in blank squares.

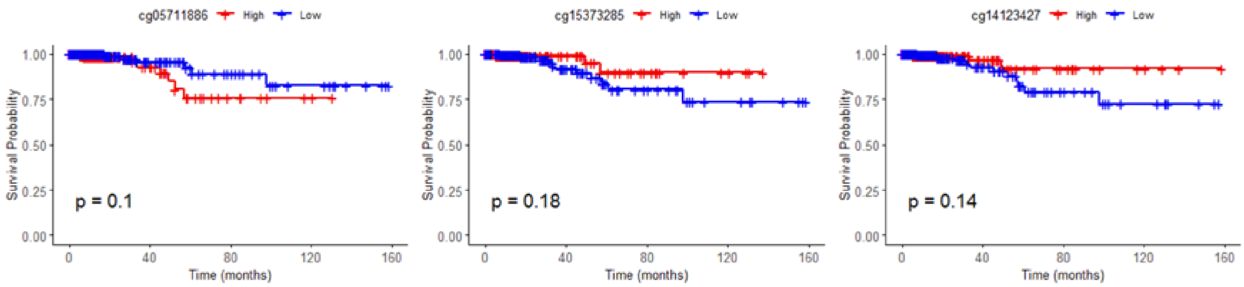

Figure S3. Kaplan Meier plots of the probes with lower p-values in THCA.

Table S3. Survival data for top 3 CpGs. Methylation-survival statistics for cg05711886, cgcg14123427, and cg15373285 for the types of cancer in which its methylation status has a significative correlation with overall survival data (likelihood-ratio test p-value < 0.05) - data retrieved from MethSurv.

Name	Cancer	LR_test_pvalue
cg05711886	LGG	0.001460388
cg05711886	KIRP	0.009810352
cg05711886	STAD	0.021432359
cg05711886	PAAD	0.02792074
cg05711886	UCEC	0.042567319
cg14123427	GBM	0.005727987
cg14123427	SKCM	0.006716647
cg14123427	KIRP	0.009303401
cg14123427	KIRC	0.029930684
cg14123427	LAML	0.035033074
cg14123427	UVM	0.049922258
cg15373285	KIRP	0.001616823
cg15373285	SKCM	0.006530816
cg15373285	KIRC	0.007460541
cg15373285	LUSC	0.011702354
cg15373285	UCEC	0.0199929
cg15373285	SARC	0.030813999
cg15373285	GBM	0.032919155
cg15373285	STAD	0.0365208
cg15373285	ESCA	0.039984993
